# Pharmacology-Informed Human Genetic Evaluation of *Panax notoginseng* Saponin-Related Candidate Genes for Functional Outcome After Ischemic Stroke Using Mendelian Randomization and Colocalization

**DOI:** 10.3390/genes17091155

**Published:** 2026-09-20

**Authors:** Guoliang Deng, Tong Cui, Yifeng Xu, Wanning Gao, Nan Li, Lijuan Jiang, Wenfeng Zhang

**Affiliations:** College of Basic Medicine, Changchun University of Chinese Medicine, No. 1035 Boshuo Road, Jingyue National High-Tech Industrial Development District, Changchun 130117, China; 17609966754@163.com (G.D.); 18743598606@163.com (T.C.); 17743170656@163.com (Y.X.); gwn9505@163.com (W.G.); 16624318273@163.com (N.L.); jianglj@ccucm.edu.cn (L.J.)

**Keywords:** Panax notoginseng saponins, ischemic stroke, functional outcome, Mendelian randomization, colocalization, KMO, cis-eQTL

## Abstract

**Background/Objectives:** Oral Xuesaitong soft capsules have been reported to improve functional outcomes after ischemic stroke, but their molecular mediators remain uncertain. We evaluated human genetic support for pharmacology-informed *Panax notoginseng* saponin-related candidate genes. **Methods:** A targeted pharmacological audit was followed by cis-QTL Mendelian randomization (MR) and regional colocalization using GISCOME outcomes. The severity-adjusted ordinal modified Rankin Scale (mRS) was primary. Eleven CKLF, KMO, and MT2A gene–tissue hypotheses formed the eQTL testing family; HMOX1 and OGA underwent pQTL analyses. A 13-test Bonferroni sensitivity calculation combined these hypotheses. Colocalization required PP.H4 ≥ 0.80. KMO analyses covered 15 GTEx tissues, three outcomes, and two external eQTL datasets. The summary-statistics study was not prospectively registered. **Results:** The audit identified 29 component–protein relations involving 19 proteins. Further, 7 of 11 eQTL hypotheses were harmonized. Higher genetically predicted KMO expression in whole blood was associated with better ordinal mRS (β = −0.906; 95% CI, −1.521 to −0.290; *p* = 0.0039; adjusted *p* = 0.043 for 11 tests and 0.051 for 13 tests). HMOX1 and OGA MR estimates were nonsignificant. Primary PP.H4 values were 0.033, 0.027, and 0.535 for HMOX1, OGA, and KMO, respectively. None of the 45 GTEx or six external comparisons met the criterion under primary priors. **Conclusions:** No candidate met the joint MR and colocalization criterion. KMO significance was sensitive to multiplicity and standard-error specification, while colocalization support was insufficient and prior-sensitive. These findings do not establish a therapeutic target or test the efficacy of oral Xuesaitong.

## 1. Introduction

Ischemic stroke remains a major cause of long-term disability. In a randomized, double-blind trial conducted at 67 centers in China, 3072 patients received oral Xuesaitong soft capsules or placebo in addition to standard care. At three months, modified Rankin Scale (mRS) scores of 0–2 occurred in 89.3% and 82.4% of the respective groups (odds ratio, 1.95; 95% confidence interval [CI], 1.56–2.44) [1]. The clinical effect motivates investigation of the molecular targets that may contribute to functional outcome after stroke.

*Panax notoginseng* saponins (PNSs) comprise parent compounds, including notoginsenoside R1 and ginsenosides Rg1, Rb1, Rd, and Re, together with oral metabolites such as compound K and 20(S)-protopanaxadiol. Gut microbial deglycosylation and oxidative metabolism influence systemic exposure [2], while measured plasma concentrations of several parent compounds are in the ng/mL range [3]. Candidate relations derived from high-concentration experiments or targeted delivery systems, therefore, require separate assessment of pharmacological plausibility and formulation-matched exposure.

Human genetic support is associated with greater success in drug development and can refine target selection [4,5,6]. Drug-target MR generally uses cis variants near an encoding gene to proxy protein abundance or gene expression, but an MR association alone may reflect linkage rather than a shared causal signal [7,8,9]. Regional colocalization provides a complementary test of whether the molecular trait and clinical outcome are consistent with the same causal variant. GISCOME supplies within-case genome-wide association summary statistics for functional status after ischemic stroke [10]. This resource has previously supported druggable-genome MR analyses [11] and a focused ABCC2 study [12].

We examined a pharmacology-informed set of PNS-related candidate genes using cis-pQTL or cis-eQTL MR, multiplicity control, and regional colocalization. The primary objective was to determine whether any evaluated candidate showed both an MR association with post-stroke mRS and PP.H4 ≥ 0.80. The analysis included all eligible GTEx tissues and three GISCOME outcome definitions for KMO, together with external eQTL evaluation and a predefined decision rule for candidate-specific transcriptomic follow-up.

## 2. Materials and Methods

### 2.1. Study Design and Reporting

**Figure 1:**
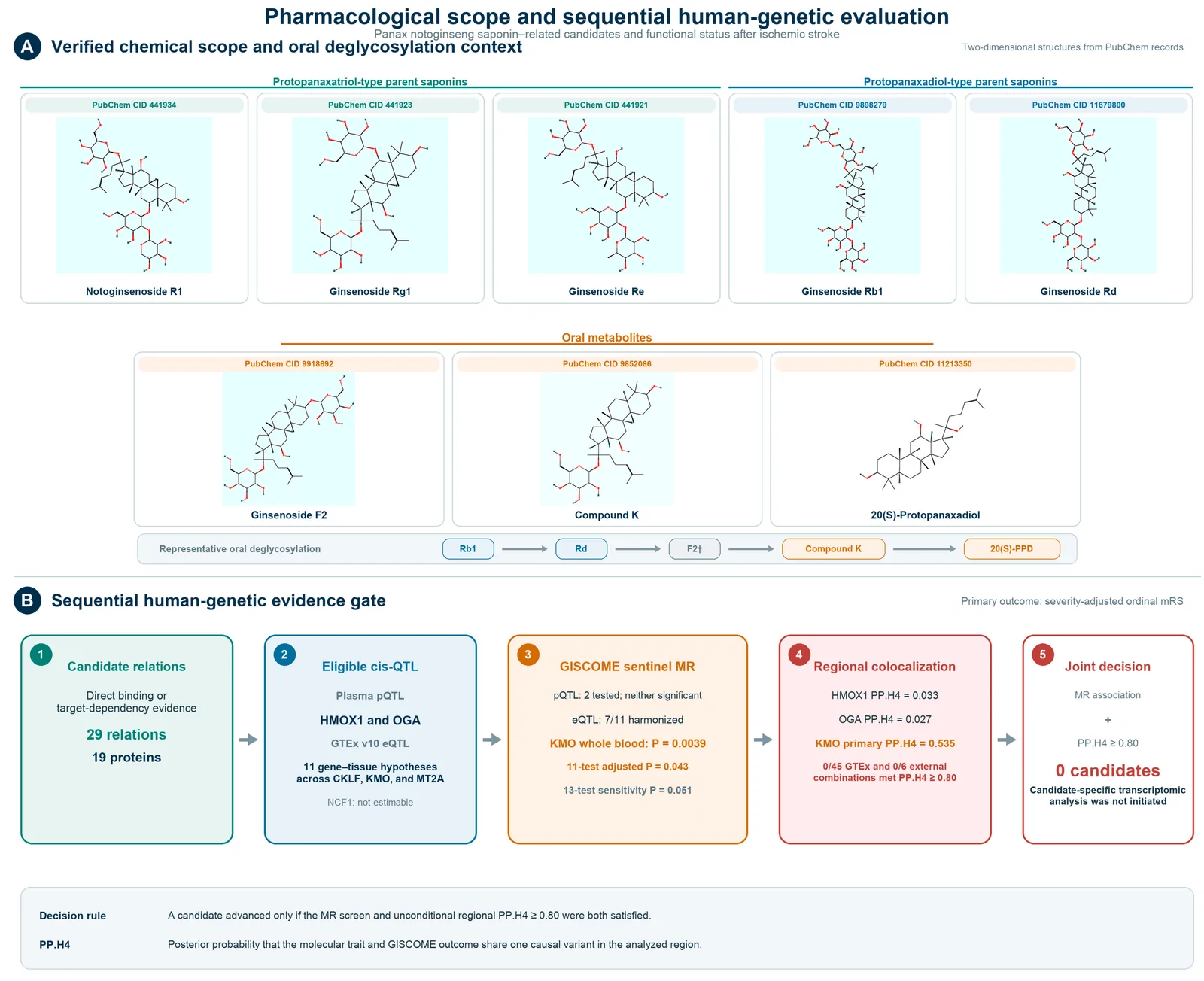
Verified chemical scope and sequential human-genetic evidence gate. (**A**) Two-dimensional structures of the five parent saponins, the two oral metabolites included in the candidate audit, and ginsenoside F2 as a pathway intermediate. Structures were generated programmatically from the following PubChem compound records: notoginsenoside R1 (CID 441934), ginsenoside Rg1 (CID 441923), ginsenoside Re (CID 441921), ginsenoside Rb1 (CID 9898279), ginsenoside Rd (CID 11679800), ginsenoside F2 (CID 9918692), compound K (CID 9852086), and 20(S)-protopanaxadiol [20(S)-PPD; CID 11213350]. The representative Rb1 oral deglycosylation sequence is Rb1 → Rd → F2 → compound K → 20(S)-PPD; †: F2 is displayed to preserve pathway continuity but was not part of the component–protein candidate audit. (**B**) Component–protein relations were evaluated sequentially according to eligible cis-QTL availability, sentinel Mendelian randomization (MR) against the severity-adjusted ordinal modified Rankin Scale (mRS) outcome from GISCOME, and regional colocalization. The KMO whole-blood association passed correction within the internally defined 11-hypothesis eQTL family but not the broader 13-hypothesis sensitivity correction under the original SE specification. The returned-SE sensitivity analysis is reported separately in Section 3.3. No candidate met the joint requirement of an MR association and unconditional regional PP.H4 ≥ 0.80; therefore, candidate-specific transcriptomic analysis was not initiated. PP.H4 denotes the posterior probability that the molecular trait and GISCOME outcome share one causal variant within the analyzed region. In panel A, green and blue distinguish the protopanaxatriol- and protopanaxadiol-type parent saponins, respectively, while orange identifies oral metabolites or the displayed pathway intermediate. In panel B, colors distinguish successive evidence stages and do not encode effect magnitude or statistical significance.

We used a sequential design comprising a candidate-relation audit, cis-QTL assessment, sentinel MR, regional colocalization, and external eQTL evaluation (Figure 1). A gene advanced to candidate-specific transcriptomic analysis only if it met both the MR and colocalization criteria. We followed STROBE-MR and established guidance for MR investigations [13,14]. The analyses used de-identified summary statistics and did not recruit participants. The archived internal decision log records analysis decisions and deviations; it is not a prospective public registration. The study was not prospectively registered. The completed reporting checklist is provided as Supplementary File S1.

### 2.2. Candidate Relations and Clinical Attainability

The candidate audit covered notoginsenoside R1; ginsenosides Rg1, Rb1, Rd, and Re; compound K; and 20(S)-protopanaxadiol. These compounds represented five parent saponins and two metabolites detected after oral administration. Candidate relations were assembled through compound-specific searches of PubMed and PubMed Central and backward citation tracing of primary mechanistic studies available through 24 August 2026. Search concepts paired each compound name with binding, target, affinity, Kd, Ki, IC50, SPR, BLI, MST, DARTS, CETSA, mutagenesis, knockdown, knockout, or rescue. Curated target databases and docking reports were used only to identify leads and did not constitute evidence for inclusion.

The unit of assessment was a unique compound–human protein pair, mapped by PubChem CID and HGNC symbol. Tier A1 required quantitative direct binding to a human protein. Tier A2 required target-specific dependency supported by binding-site mutagenesis, target perturbation, rescue, or a comparably specific functional experiment when a directly comparable affinity estimate was unavailable. Database prediction alone, docking, disease–gene overlap, pathway membership, and downstream expression change were excluded. Duplicate reports of the same pair were consolidated at the highest supported tier. This procedure yielded 29 relations (19 A1 and 10 A2) involving 19 unique proteins. The assay, species, potency or affinity, exposure assessment, decision, and source for each relation are reported in Table S1 and the archived machine-readable audit file. Representative relations included Rd-SLC5A1 [15], Rg1-CKLF [16], Rb1-HMOX1 [17], and compound K-OGA [18]. This targeted audit defined the pharmacologically anchored candidate set and was not a systematic review.

We defined clinical attainability as formulation-matched human unbound exposure that could be compared with Kd, IC50, or EC50 on the same molar scale. No such data were available for oral Xuesaitong soft capsules, so clinical attainability remained unresolved.

### 2.3. QTL and Outcome Data

Plasma pQTL coverage was assessed using UK Biobank Pharma Proteomics Project data (2923 proteins; *n* = 54,219) [19] and deCODE SomaScan data (4907 aptamers; *n* = 35,559) [20]. Formal pQTL colocalization used complete European-ancestry UKB-PPP regional statistics (Table S2). Proteins without an eligible pQTL were assessed for cis-eQTL coverage in GTEx v10. KMO eGene status was audited across all 54 tissues; all 15 eGene tissues were included. External KMO eQTL analyses used BLUEPRINT CD14+ monocytes and BrainSeq dorsolateral prefrontal cortex data from the eQTL Catalogue [21,22,23,24]. Complete KMO eGene coverage across all 54 GTEx v10 tissues is reported in Table S7.

The QTL analyses were organized by molecular exposure and data source, not by biological gene family. HMOX1 and OGA had eligible cis-pQTLs and were evaluated as plasma protein abundance exposures. CKLF, KMO, and MT2A lacked an eligible protein instrument in the audited resources and were evaluated through 11 GTEx gene–tissue hypotheses. These expression-based hypotheses shared a selection procedure and formed the primary Bonferroni family. Protein- and expression-QTL estimates were not pooled because they proxy different molecular traits. Separate analysis of these traits does not, however, resolve the study-wide multiplicity question. We, therefore, also report a broader 13-test Bonferroni sensitivity calculation comprising the 11 eQTL hypotheses and the 2 estimable pQTL analyses. This calculation assesses the sensitivity of the KMO finding to the testing family; it is not a correction over all 19 pharmacological candidates. Three HMOX1 brain-tissue eQTL analyses were supportive checks and did not determine candidate advancement; they were outside both testing families. NCF1 was not estimable and was not included in the 13-test sensitivity denominator.

GISCOME outcomes comprised severity-adjusted ordinal mRS (*n* = 6021), severity-adjusted mRS 0–2 versus 3–6 (*n* = 6021), and severity-adjusted mRS 0–1 versus 2–6 (*n* = 4363) [10]. In the source GWAS, each cohort fitted an additive-genetic multivariable model adjusted for age, sex, ancestry (up to five principal components, according to cohort availability), and baseline stroke severity measured by the NIHSS 0–10 days after stroke, with preference for assessment near day 0 or 1. The two binary outcomes were analyzed via logistic regression in PLINK v1.90b4.6, whereas the full mRS was analyzed by cumulative-logit ordinal regression using the MATLAB R2016b (Statistics and Machine Learning Toolbox; MathWorks, Natick, MA, USA) mnrfit algorithm. The same covariate set was used for the adjusted binary and ordinal models, and cohort-specific estimates were combined by inverse-variance fixed-effect meta-analysis. We also evaluated ordinal mRS without baseline-NIHSS adjustment as a sentinel-MR sensitivity outcome; it was not included in the 45-comparison regional matrix.

Severity-adjusted ordinal mRS was selected as the primary outcome because it estimates follow-up disability conditional on the initial neurological deficit and retains information from all seven mRS categories [25,26]. This adjustment changes the estimand: if a candidate influences initial stroke severity, conditioning on NIHSS may remove part of its total association with later disability or introduce bias. Accordingly, the outcome is described as post-stroke functional status, not recovery or change from baseline. mRS was assessed 60–190 days after stroke, with approximately 80% of assessments at three months +/− two weeks; the source GWAS did not model a baseline-to-follow-up mRS change score, and baseline NIHSS entered only as a covariate [10].

### 2.4. Instrument Selection, Harmonization, and MR

Cis windows were defined explicitly for each QTL layer. For the UKB-PPP HMOX1 and OGA pQTL analyses, variants were extracted from the protein-coding gene boundaries plus 1,000,000 bp on each side: HMOX1, chr22:34,381,096-36,394,207 in GRCh38 and chr22:34,777,089-36,790,200 in GRCh37; OGA, chr10:100,784,450-102,818,444 in GRCh38 and chr10:102,544,207-104,578,201 in GRCh37. For GTEx v10 sentinel eQTL selection, cis was defined by the GTEx single-tissue convention as the transcription start site plus or minus 1,000,000 bp in GRCh38 for the relevant gene–tissue pair. Formal KMO regional colocalization across all GTEx tissues and all three GISCOME outcomes used the KMO transcription start site at chr1:241,532,134 (GRCh38), giving the inclusive interval chr1:240,532,134-242,532,134. BLUEPRINT monocyte and BrainSeq dorsolateral prefrontal cortex analyses were restricted to the same KMO interval after coordinate harmonization.

Harmonization procedures differed by analysis layer. For pQTL regional inputs, variants were matched by position and alleles, allowing strand complements; the mapping with the smallest effect-allele-frequency difference was selected, and differences > 0.20 were excluded. Sentinel eQTL matching used rsIDs and direct or reverse–complement allele pairs. Regional GTEx eQTL analyses used build-harmonized positions and allele sets, restricted to simple SNPs with the GTEx MAF ≥ 1% flag. The sentinel eQTL workflow did not apply a separate cross-dataset allele-frequency filter or an explicit palindromic-variant exclusion. Instrument strength was summarized by the F statistic, calculated as the squared ratio of the exposure effect to its standard error. For sentinel eQTLs, exposure effects were the GTEx normalized effect size (NES); standard errors were approximated from |NES| divided by the standard-normal quantile corresponding to the two-sided eQTL *p* value. Single-variant effects used the Wald ratio [27,28,29]. The pQTL screening estimates used first-order standard errors, SE(outcome)/|β(exposure)|. The eQTL estimates used a second-order delta-method variance, incorporating uncertainty in both exposure and outcome effects, under the working assumption of negligible covariance between those estimates. Native ordinal coefficients were multiplied by −1 and binary coefficients by +1 so that positive MR estimates represented worse mRS. For eQTL MR, estimates are on the outcome log-odds scale per unit of normalized expression, not changes in mRS points.

Eleven CKLF, KMO, and MT2A gene–tissue hypotheses constituted the internally defined multiplicity family. For each hypothesis, we queried the GTEx v10 significant single-tissue eQTL endpoint for the exact GENCODE gene and tissue and selected one exposure-side sentinel: the variant with the smallest nominal *p* value. If several records had an identical minimum *p* value, the variant with the lowest GRCh38 position and then the lexicographically first variant identifier was selected. The endpoint returned 2, 29, 2, 51, 46, 3, 4, 23, 2, 4, and 2 significant variant–gene pairs for CKLF–caudate; KMO–whole blood, caudate, cortex, frontal cortex, nucleus accumbens, and coronary artery; and MT2A–whole blood, caudate, aorta, and tibial artery, respectively. The saved responses contained all entries reported by their pagination metadata. These are counts of significant variant–gene pairs, not conditionally independent cis-eQTL signals. Independent signal counts were not established in this single-sentinel analysis. Thus, one eligible sentinel was carried forward for each of the 11 hypotheses; seven were present and harmonizable in GISCOME; and four were absent. No outcome-side substitution was permitted. The primary Bonferroni correction used all 11 hypotheses, including variants absent from GISCOME; the broader sensitivity calculation multiplied the unadjusted *p* value by 13, capped at 1; Benjamini–Hochberg q values across evaluable tests were descriptive. Reverse MR was not performed because no independent strong outcome-side instrument was available in the candidate regions. The full hypothesis-level record is provided in Table S3.

Sensitivity checks compared the ordinal outcome with and without baseline-NIHSS adjustment. A post hoc input-consistency check also recalculated the primary KMO whole-blood Wald estimate using the standard error returned in the regional GTEx response instead of the normal-approximation sentinel standard error. The original sentinel analysis was retained as primary; this check evaluated dependence on the standard-error specification.

### 2.5. Regional Colocalization

HMOX1 and OGA pQTL regions extended 1 Mb on either side of each protein-coding gene. KMO was defined by the GENCODE v39/GRCh38 transcription start site at chr1:241,532,134, with a standard TSS ± 1 Mb region. Variants were matched by position and biallelic allele set, allowing strand complements. We used a Python implementation of the coloc approximate-Bayes-factor model, which assumes at most one causal variant for each trait within an analyzed region, to estimate PP.H0 (neither trait associated), PP.H1 (molecular trait only), PP.H2 (outcome only), PP.H3 (both traits associated through distinct causal variants), and PP.H4 (both traits associated through one shared causal variant) [30]. The primary per-variant priors, p1 = p2 = 1 × 10^−4^ and p12 = 1 × 10^−5^, were the conventional coloc defaults, with p1 and p2 denoting per-variant association priors for the molecular trait and outcome and p12 denoting the per-variant prior of association with both traits. These per-variant priors are distinct from the region-level prior probabilities of H0–H4. Because p12 can materially influence PP.H4, it was varied from 1 × 10^−6^ to 1 × 10^−4^, with 5 × 10^−6^ designated as a conservative robustness benchmark. Unconditional PP.H4 ≥ 0.80 was the prespecified colocalization criterion; PP.H3 and H4/(H3 + H4) were reported as diagnostics and could not override that criterion.

For KMO, the primary comparison was whole-blood eQTL versus severity-adjusted ordinal mRS. Regional eQTL effects and standard errors were obtained from the GTEx dynamic eQTL response, rather than reconstructed from *p* values as in the sentinel screen. Analyses included 15 eGene tissues and three outcomes (45 combinations). The primary normal effect-size prior standard deviation was 0.15 for molecular effects and ordinal-outcome coefficients, and 0.20 for binary-outcome log-odds coefficients. The ordinal specification is a working prior on the reported regression-coefficient scale, not an assertion that mRS is a standardized continuous phenotype. HMOX1 and OGA used 0.15 for both molecular and ordinal-outcome effects. Their sensitivity analyses varied the outcome prior standard deviation over 0.10, 0.15, 0.20 and 0.30. KMO GTEx and external analyses additionally varied the eQTL prior standard deviation over 0.10, 0.15 and 0.20; GTEx analyses also used TSS ± 250, 500 and 1000 kb windows. The p12 grid was 1 × 10^−6^, 5 × 10^−6^, 1 × 10^−5^, 5 × 10^−5^ and 1 × 10^−4^, with 5 × 10^−6^ used as a conservative robustness benchmark [31]. The upper-bound p12 = 1 × 10^−4^ analysis was a stress test and did not determine candidate status.

### 2.6. External eQTL Evaluation and Index-Event Bias

BLUEPRINT monocytes (*n* = 191) and BrainSeq dorsolateral prefrontal cortex (*n* = 479) were analyzed across the same KMO region and three GISCOME outcomes. Directional agreement was distinguished from regional colocalization. Because GISCOME included stroke cases, candidate instruments were also examined in the European-ancestry MEGASTROKE ischemic-stroke occurrence GWAS (GCST006908) [32,33,34]. This lookup was used to identify susceptibility associations that could indicate index-event bias; it was not a formal correction.

### 2.7. Statistical Decision Rule

We prioritized a candidate only when it had an eligible cis-QTL, an MR association after the relevant multiplicity correction, and regional PP.H4 ≥ 0.80 that remained supported under reasonable priors. Candidate-specific single-cell or bulk transcriptomic analysis was contingent on this joint criterion. Any candidate-specific single-cell follow-up would require biological-replicate-level inference to limit pseudoreplication [35,36].

### 2.8. Software and Reproducibility

Analyses used Python 3.12.13 and pysam 0.23.3. Colocalization was implemented in Python using estimate-based approximate Bayes factors and log-space aggregation of H0–H4; the analysis scripts did not invoke the R coloc package. For each variant, V was the squared standard error, W the squared effect-size prior standard deviation, z = β/SE and r = W/(V + W); the log Bayes factor was 0.5 × [log(1 − r) + r × z^2^]. Separate validation scripts recomputed posterior probabilities from harmonized inputs and compared them with saved tables. Agreement checks assess numerical consistency and do not validate trait-scale assumptions or harmonization. The reproducibility archive contains the code, parameters, derived results, checksums and figure data.

## 3. Results

### 3.1. Candidate Audit and cis-QTL Availability

The audit yielded 29 component–protein relations (19 A1 and 10 A2) involving 19 unique proteins and seven parent compounds or metabolites after mapping and deduplication by PubChem CID and HGNC symbol. None had formulation-matched unbound exposure data. Among 18 nuclear-encoded proteins, HMOX1, OGA, and NCF1 had eligible plasma cis-pQTL coverage. CKLF, KMO, and MT2A comprised the eQTL analysis family. Table 1 summarizes the principal candidates retained for genetic analysis; Table S1 reports the complete pharmacological audit.

### 3.2. pQTL MR and Colocalization

HMOX1 rs2071747 (F = 97.30) and OGA rs3781299 (F = 269.28) were harmonized with GISCOME. Neither Wald estimate was associated with severity-adjusted ordinal mRS: HMOX1 β = 0.200 (95% CI, −0.894 to 1.295; *p* = 0.720) and OGA β = 0.248 (95% CI, −0.316 to 0.812; *p* = 0.390). None of the five audited NCF1 candidate variants was found in GISCOME, so no NCF1 MR estimate was obtained.

Regional analyses retained 7144 HMOX1 and 4107 OGA variants. For HMOX1, PP.H0–H4 were 5.79 × 10^−17^, 0.631, 3.08 × 10^−17^, 0.336, and 0.033, respectively. For OGA, the corresponding values were 2.58 × 10^−54^, 0.789, 5.99 × 10^−55^, 0.183, and 0.027. For both regions, H1 had the largest posterior probability, favoring an association with the molecular trait alone. Conditional on both traits being associated, H3 was more strongly supported than H4. These results, therefore, should not be summarized as definitive evidence of distinct causal variants. The PP.H4 maxima across routine p12 ≤ 1 × 10^−5^ sensitivity analyses were 0.041 and 0.037, respectively. Neither region met the colocalization criterion (Table 2). Regional profiles are shown in Figure S1, and the complete posterior probabilities are provided in Table S4.

### 3.3. eQTL Sentinel MR

Thus, 7 of 11 CKLF/KMO/MT2A sentinel hypotheses were harmonized with GISCOME. The KMO whole-blood instrument rs3819976 had F = 36.93. Genetically predicted higher KMO expression was associated with better severity-adjusted ordinal mRS (β = −0.906; 95% CI, −1.521 to −0.290; *p* = 0.0039; Bonferroni-adjusted *p* = 0.043). This was the only association meeting correction across the 11-hypothesis eQTL family. In the broader 13-test Bonferroni sensitivity calculation, the adjusted *p* value was 0.051. The association, therefore, did not meet the 0.05 threshold under the broader correction, although its direction and effect estimate were unchanged. Cortex and frontal cortex BA9 estimates had concordant directions but used sentinels in complete linkage disequilibrium with the whole-blood sentinel and were not independent replications. F statistics ranged from 16.19 to 42.84 across the 11 selected eQTL sentinels. None of the seven harmonized primary-family sentinels was palindromic.

The KMO whole-blood estimate remained directionally consistent without baseline-NIHSS adjustment (β = −0.841; 95% CI, −1.425 to −0.256; *p* = 0.00481). Applying the same 11-hypothesis correction to this sensitivity outcome gave *p* = 0.0529. The adjusted binary-outcome estimates were also directionally consistent (mRS 0–2 versus 3–6: β = −0.897, *p* = 0.0299; mRS 0–1 versus 2–6: β = −1.119, *p* = 0.0133; both *p* values unadjusted). These overlapping outcome analyses were sensitivity comparisons, not independent replications.

In the post hoc standard-error check, the GTEx-returned KMO exposure SE was 0.065989, compared with 0.066856 from the normal approximation. The MR estimate was essentially unchanged (β = −0.906), while its SE changed from 0.314039 to 0.313126. The resulting *p* value was 0.00382, corresponding to adjusted *p* = 0.0420 for 11 tests and 0.0497 for 13 tests. The crossing of 0.05 after this small change underscores the borderline nature of the broader-family result and does not resolve the insufficient colocalization support.

The three supportive HMOX1 eQTL analyses showed no nominal association with the primary outcome (*p* = 0.508 for cortex, 0.637 for frontal cortex BA9, and 0.477 for nucleus accumbens). The frontal-cortex sentinel rs114316959 was palindromic and lacked a cross-dataset frequency check, limiting directional interpretation. The archived sentinel table includes all 56 gene–tissue–outcome records, including unavailable variants.

### 3.4. KMO Regional and External Colocalization

**Figure 2:**
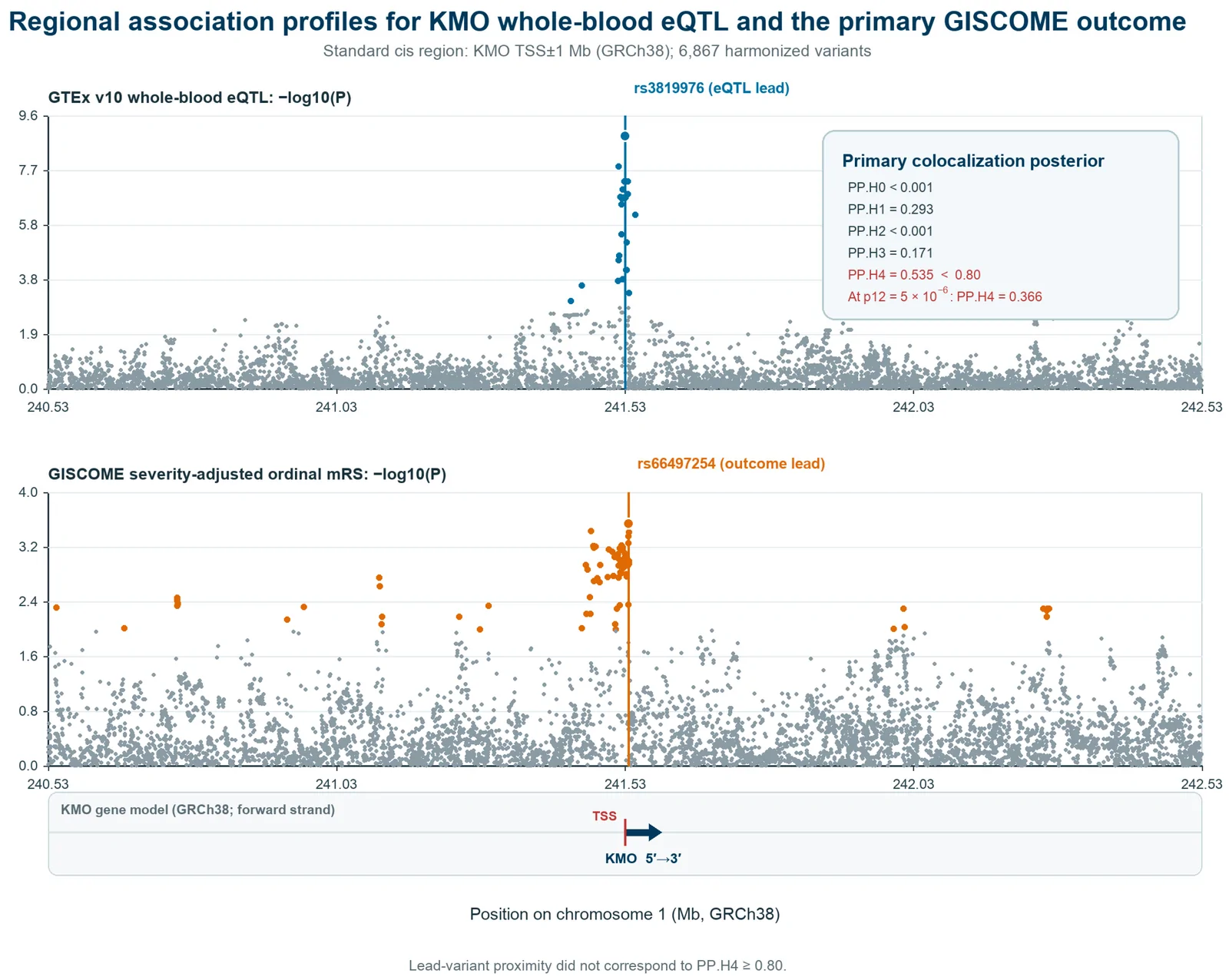
Regional association profiles for the GTEx v10 KMO whole-blood eQTL and GISCOME severity-adjusted ordinal mRS within the standard TSS ± 1 Mb region. The primary colocalization posterior was PP.H4 = 0.535. Blue points indicate eQTL *p* ≤ 0.001 and orange points indicate outcome *p* ≤ 0.01; other points are gray. Point size follows the same display thresholds. Colors do not encode linkage disequilibrium or causal status. Vertical lines mark the lead variant in each panel separately; the lower track marks the KMO TSS and transcriptional direction in GRCh38.

**Figure 3:**
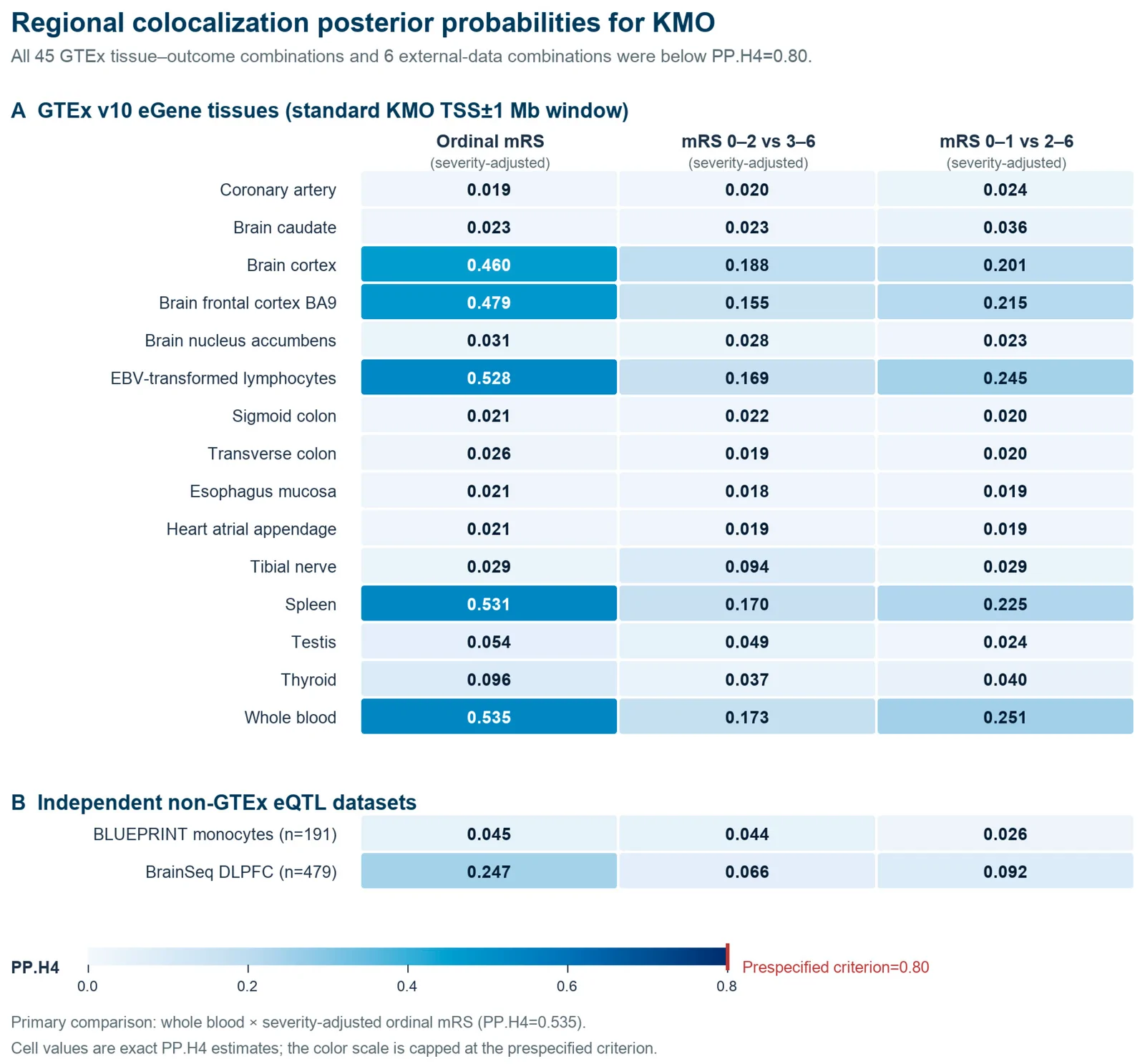
Regional colocalization posterior probabilities for KMO. Panel (**A**) shows 15 GTEx v10 eGene tissues across three GISCOME outcomes; Panel (**B**) shows BLUEPRINT monocytes and BrainSeq dorsolateral prefrontal cortex. All PP.H4 values were below 0.80.

The standard KMO cis region contained 6867 harmonized variants for whole blood and severity-adjusted ordinal mRS (Figure 2). The eQTL and outcome lead variants were separated by 5.9 kb. In this primary comparison, PP.H0–H4 were 0.000269, 0.292689, 0.000158, 0.171475, and 0.535409, respectively. H4 was the most strongly supported individual hypothesis, but substantial posterior probability remained on H1 and H3. Thus, support for a shared causal variant was inconclusive under the PP.H4 ≥ 0.80 criterion; the result did not establish either a shared signal or distinct causal variants. All 45 GTEx tissue–outcome combinations had PP.H4 < 0.80. For ordinal mRS, the highest values were observed in whole blood (0.535), spleen (0.531), EBV-transformed lymphocytes (0.528), frontal cortex BA9 (0.479), and cerebral cortex (0.460) (Figure 3). The maxima for the two binary outcomes were 0.188 and 0.251. The complete 45-comparison matrix is provided in Table S5.

For the primary whole-blood comparison, PP.H4 decreased to 0.366 at p12 = 5 × 10^−6^. The maximum across the expanded GTEx sensitivity grid at this prior was 0.593. All six external eQTL–outcome combinations had PP.H4 < 0.80; the maximum was 0.247 for BrainSeq cortex versus ordinal mRS, and the expanded-grid maximum at p12 = 5 × 10^−6^ was 0.347. Separate posterior recomputation agreed to within 1 × 10^−12^. Across the GTEx grid with p12 ≤ 1 × 10^−5^, the maximum PP.H4 was 0.744. At p12 = 1 × 10^−4^ and the maximum increased to 0.967 in brain cortex versus ordinal mRS, using TSS ± 250 kb and effect-size prior SDs of 0.20 and 0.30 for expression and outcome, respectively. This permissive-prior stress test was disclosed but did not determine candidate advancement. The complete prior, window, and effect-size sensitivity grid is reported in Table S8. The six external combinations and numerical-validation checks are reported in Tables S6 and S10, respectively.

### 3.5. Stroke-Susceptibility Lookup and Final Decision

In the MEGASTROKE European-ancestry ischemic-stroke susceptibility GWAS, the effect-allele log-odds estimates were β = 0.0121 (SE = 0.0109; *p* = 0.267) for OGA rs3781299, β = 0.0644 (SE = 0.0219; *p* = 0.00323; Bonferroni-adjusted *p* = 0.00969) for HMOX1 rs2071747, and β = −0.0128 (SE = 0.0337; *p* = 0.705) for KMO rs3819976. The HMOX1 association heightened concern that conditioning on ischemic-stroke case status could distort its within-case functional-outcome estimate. The null KMO lookup provided no evidence that its sentinel affected stroke susceptibility in this dataset, but it did not exclude index-event bias through other variants, pathways, or limited power and was not a formal bias correction. The KMO MR estimate was, therefore, retained as a within-case association requiring cautious interpretation, rather than as an index-event-bias-corrected causal effect. No candidate met the joint MR and colocalization criterion, so no candidate-specific transcriptomic analysis was undertaken. The complete susceptibility lookup is reported in Table S9.

## 4. Discussion

### 4.1. Principal Findings

The evaluated candidates did not meet the joint MR and colocalization criterion, but the reasons differed. NCF1 could not be estimated because its instrument was absent from GISCOME. HMOX1 and OGA had nonsignificant MR estimates and regional posteriors dominated by molecular-trait-only association. KMO whole-blood expression showed a single-sentinel association that met correction within the 11-test eQTL family but not the broader 13-test sensitivity correction. Its regional evidence was insufficient for a confident shared-variant assignment.

The principal uncertainty concerns the interpretation of the KMO association. The 6867-variant whole-blood analysis favored H4 over each alternative hypothesis individually (PP.H4 = 0.535), but support did not reach 0.80 and fell to 0.366 under the conservative shared-variant prior. None of the 45 GTEx tissue–outcome combinations or 6 external eQTL–outcome combinations met the criterion under the primary priors. These comparisons assess consistency across molecular datasets and outcome definitions; they are not 51 independent replications because they reuse GISCOME outcomes and include correlated tissues and nested outcome definitions. The findings, therefore, support retaining KMO as an unresolved pharmacological hypothesis rather than prioritizing it as a genetically supported target or concluding that it has no biological role.

### 4.2. Biological Interpretation

KMO catalyzes the conversion of kynurenine to 3-hydroxykynurenine, directing metabolism toward a branch that can subsequently produce quinolinic acid. Studies in stroke and other neurological settings link this pathway to excitotoxicity, oxidative stress, and immune responses, with marked dependence on tissue and disease context [42,43,44]. KMO inhibition reduced post-ischemic neuronal injury in experimental systems [45], and lower KMO expression accompanied improved recovery in a mouse study of circSCMH1 [46]. In our analysis, however, genetically predicted higher KMO expression was associated with better mRS. Because gene expression, enzyme activity, tissue context, and treatment timing are not equivalent, this direction does not support a simple KMO inhibition mechanism.

Compound K binds KMO and produced KMO-dependent pharmacological effects in a depression model [41]. Its relevance to oral Xuesaitong remains uncertain because formulation-matched unbound exposure data are unavailable. Further evaluation of KMO would need regional genetic replication and pharmacokinetic evidence consistent with the proposed intervention. A binding or target-dependency experiment does not establish that the drug effect is equivalent to the lifelong expression difference proxied by a whole-blood eQTL. A mechanistic interpretation would require agreement on the relevant cell type, the direction of enzyme modulation, and exposure at the target site.

Higher serum HMOX1 has been associated with better three-month outcomes after ischemic stroke [38]. In our pQTL analysis, HMOX1 had low PP.H4, and its susceptibility association could bias a within-case estimate. OGA is relevant to cerebral O-GlcNAcylation and ischemia–reperfusion injury [39], but the compound K–OGA evidence came from a brain-targeted liposomal formulation [18]. These findings provide biological context without establishing either gene as a genetically supported target. For both HMOX1 and OGA, the genetic exposure was plasma protein abundance; this should not be assumed to represent intracellular enzyme activity in the cells mediating ischemic injury.

### 4.3. Methodological Considerations

The shared-variant prior materially affected the KMO posterior: PP.H4 decreased from 0.535 in the primary analysis to 0.366 at the conservative p12 benchmark. For HMOX1 and OGA, most posterior mass favored a molecular-trait-only signal or distinct signals. Our colocalization model allowed one causal variant per trait. Multi-signal approaches such as coloc-SuSiE could improve resolution when validated, ancestry-matched linkage-disequilibrium data become available [47,48].

Because GISCOME includes only people who had an ischemic stroke, its estimates may be affected by index-event bias [32,33]. The MEGASTROKE lookup identified a susceptibility association for the HMOX1 instrument, although this lookup cannot quantify or correct the bias. A nonsignificant susceptibility lookup for KMO likewise does not establish freedom from selection bias.

### 4.4. Strengths and Limitations

The analysis used a clinically aligned functional outcome, complete KMO eGene-tissue coverage, three outcome definitions, external eQTL datasets, prior sensitivity analyses, and a separate posterior calculation. Several constraints limit interpretation. The candidate audit was targeted rather than systematic; formulation-matched unbound exposure and QTL coverage were incomplete; GISCOME had a modest sample size and variable mRS assessment timing; and the genetic datasets were predominantly European, whereas the clinical trial was conducted in China. Possible sample overlap and index-event bias also remain.

Because the primary GISCOME outcome was conditioned on baseline NIHSS, the estimates concern follow-up mRS conditional on initial neurological severity rather than the total genetic association with outcome; conditioning may also introduce bias when a candidate affects initial severity. The KMO association was sensitive to both the multiplicity family and the standard-error specification. The 13-test sensitivity calculation included the two estimable pQTL analyses but was not a study-wide correction across all pharmacological candidates. Sentinel eQTL standard errors were reconstructed using a normal approximation, whereas regional GTEx analyses used returned standard errors; the post hoc KMO input-consistency check showed that this small SE difference was sufficient to move the 13-test-adjusted *p* value across 0.05. Sentinel eQTL harmonization did not include explicit palindromic-variant exclusion or cross-dataset frequency filtering. None of the seven harmonized primary-family sentinels was palindromic, but the supportive HMOX1 frontal-cortex estimate retained this ambiguity. The ordinal-outcome effect-size prior was a working approximation; prior sensitivity analyses assess dependence on this choice but do not validate the underlying scale.

Single-variant MR does not permit conventional pleiotropy tests, and single-signal colocalization cannot resolve allelic heterogeneity. Selecting and estimating the strongest eQTL in the same GTEx sample may also introduce exposure-side winner’s curse, which can inflate the estimated SNP–expression association and tend to attenuate the magnitude of a Wald ratio estimate; the KMO tissue-specific sentinels may additionally tag correlated rather than independent regulatory signals. Finally, lifelong genetically proxied molecular differences do not reproduce short-term treatment with a multicomponent formulation.

## 5. Conclusions

None of the evaluated PNS-related candidates met the joint MR and regional colocalization criterion for post-stroke functional status. The KMO single-sentinel association was sensitive to the multiplicity family, and shared-variant support remained inconclusive and prior-sensitive. HMOX1 and OGA showed no clear MR support, whereas NCF1 was not estimable. These findings limit genetic target prioritization from the available data; they do not exclude biological involvement or test the clinical efficacy of oral PNS. Further work should resolve the regional genetic signal and establish whether the relevant pharmacological exposure and molecular perturbation are represented by the genetic proxy.

## Figures and Tables

**Table 1:** Principal PNS-related candidates retained for genetic analysis.

Candidate	Pharmacological Basis, Principal Function, and Human Disease Relevance	QTL Evidence	Genetic Result	Interpretation
HMOX1	Rb1 binding in a sepsis model [17]; inducible heme degradation and oxidative-stress response; HMOX1 deficiency [37] and serum HO-1 association with ischemic-stroke outcome [38]	UKB-PPP cis-pQTL	MR *p* = 0.720; PP.H4 = 0.033	No joint support
OGA	Compound K binding in a targeted-liposome stroke model [18]; removal of O-GlcNAc from intracellular proteins; O-GlcNAc dysregulation is relevant to ischemia–reperfusion injury [39] and metabolic or neurodegenerative disease	UKB-PPP cis-pQTL	MR *p* = 0.390; PP.H4 = 0.027	No joint support
NCF1	Rb1–p47phox relation; organizer subunit of the phagocyte NADPH oxidase; biallelic NCF1 deficiency causes autosomal-recessive chronic granulomatous disease [40]	Complex locus	Five audited candidate variants absent from GISCOME	Not estimable
KMO	Compound K binding [41]; mitochondrial conversion of kynurenine to 3-hydroxykynurenine; kynurenine-pathway abnormalities are reported in stroke and neurodegenerative or neuropsychiatric disorders [42,43,44]	15 GTEx eGene tissues; 2 external datasets	MR *p* = 0.0039; adjusted *p* = 0.043 (11 tests) and 0.051 (13-test sensitivity); primary PP.H4 = 0.535	Multiplicity- and SE-sensitive MR association; insufficient colocalization support

Note: Human disease relevance summarizes established deficiency phenotypes or reported clinical/pathway associations; it does not imply that the present MR and colocalization analyses support the candidate as a therapeutic target. Abbreviations: MR, Mendelian randomization; PP.H4, posterior probability of a shared causal variant.

**Table 2:** Primary regional colocalization results under p1 = p2 = 1 × 10^−4^ and p12 = 1 × 10^−5^.

Region and Exposure	PP.H1	PP.H3	PP.H4	PP.H4 ≥ 0.80 ?
HMOX1 plasma pQTL	0.631	0.336	0.033	No
OGA plasma pQTL	0.789	0.183	0.027	No
KMO whole-blood eQTL	0.293	0.171	0.535	No

Note: All rows report the primary severity-adjusted ordinal mRS analysis; the KMO row refers to whole blood. PP.H1 represents association with the molecular trait alone, PP.H3 association with both traits through distinct causal variants, and PP.H4 a shared causal variant. Complete H0–H4 probabilities are given in Section 3.2 and Section 3.4. No primary comparison reached PP.H4 ≥ 0.80. For the same KMO whole-blood comparison, lowering p12 to 5 × 10^−6^ reduced PP.H4 from 0.535 to 0.366. Maxima across tissues, outcomes, windows, or effect-size priors are reported separately in Section 3.4 and are not paired estimates of prior sensitivity.

## Data Availability

GISCOME, UKB-PPP, deCODE, GTEx v10, eQTL Catalogue, and MEGASTROKE data were obtained from their originating repositories under the applicable access conditions. Source locations, code, parameters, derived results, checksums, and figure data, together with the key machine-readable file inventory (Table S11), are deposited in a Zenodo draft with reserved DOI https://doi.org/10.5281/zenodo.22124922. Private reviewer access will be provided through the submission system, and the record will be released publicly no later than article publication. OpenAI Codex assisted with language editing, analysis-code development and debugging, and organization of figures and supporting files. Computational checks included reconciliation with saved results, allele-alignment checks, and independent recalculation of posterior probabilities. These checks did not replace scientific review by the authors, who take responsibility for the study and its final content.

## Supplementary Materials

The following supporting information can be downloaded at: https://www.mdpi.com/article/10.3390/genes17091155/s1, Figure S1: Regional association profiles for HMOX1 and OGA pQTLs and GISCOME severity-adjusted ordinal mRS; Table S1: Twenty-nine A1/A2 component–protein relations; Table S2: pQTL/eQTL coverage for 18 nuclear-encoded candidates; Table S3: Eleven analysis-plan-defined gene–tissue hypotheses; Table S4: Primary regional posterior probabilities for HMOX1 and OGA; Table S5: KMO regional colocalization across 15 tissues and three outcomes; Table S6: Six BLUEPRINT and BrainSeq colocalization combinations; Table S7: KMO eGene status in all 54 GTEx v10 tissues; Table S8: Summary of colocalization sensitivity analyses; Table S9: Candidate instruments and ischemic-stroke occurrence risk in European ancestry; Table S10: Numerical validation and data integrity; Table S11: Key machine-readable files; Supplementary File S1: Completed STROBE-MR checklist [49]. The supporting information includes the complete component–protein audit, cis-QTL coverage, all 11 primary-family sentinel hypotheses, the three supportive HMOX1 tissue analyses and the ordinal sensitivity outcome without NIHSS adjustment, HMOX1 and OGA regional results, all 45 GTEx and six external KMO colocalization combinations, the 54-tissue eGene audit, prior-sensitivity results, the MEGASTROKE lookup, posterior recomputation checks, and the STROBE-MR checklist. Machine-readable results and code are included in the reproducibility archive.

## Abbreviations

ABF, approximate Bayes factor; BA9, Brodmann area 9; BLI, biolayer interferometry; CETSA, cellular thermal shift assay; CI, confidence interval; CID, PubChem compound identifier; DARTS, drug affinity responsive target stability; EBV, Epstein–Barr virus; EC50, half-maximal effective concentration; eQTL, expression quantitative trait locus; GISCOME, Genetics of Ischaemic Stroke Functional Outcome consortium; GENCODE, Encyclopedia of DNA Elements gene annotation; GRCh37/GRCh38, Genome Reference Consortium Human Build 37/38; GTEx, Genotype-Tissue Expression project; GWAS, genome-wide association study; LD, linkage disequilibrium; HGNC, HUGO Gene Nomenclature Committee; IC50, half-maximal inhibitory concentration; Kd, equilibrium dissociation constant; Ki, inhibition constant; LC–MS/MS, liquid chromatography–tandem mass spectrometry; MAF, minor allele frequency; MR, Mendelian randomization; mRS, modified Rankin Scale; MST, microscale thermophoresis; NES, normalized effect size; NIHSS, National Institutes of Health Stroke Scale; O-GlcNAc, O-linked β-N-acetylglucosamine; PNS, *P. notoginseng* saponins; PPD, protopanaxadiol; PP.H0–PP.H4, posterior probabilities for the five colocalization hypotheses; pQTL, protein quantitative trait locus; QTL, quantitative trait locus; SE, standard error; SNP, single-nucleotide polymorphism; SPR, surface plasmon resonance; STROBE-MR, Strengthening the Reporting of Observational Studies in Epidemiology Using Mendelian Randomization; TSS, transcription start site; UKB-PPP, UK Biobank Pharma Proteomics Project.
