# Supplementary material for: Pharmacology-Informed Human Genetic Evaluation of Panax notoginseng Saponin-Related Candidate Genes for Functional Outcome After Ischemic Stroke Using Mendelian Randomization and Colocalization
Source: Genes (Basel). 2026 Sep 20;17(9):1155. doi: 10.3390/genes17091155 (PMC13606239; doi:10.3390/genes17091155)
Supplement: Supplementary file 1 [file genes-17-01155-s001.zip › Supplementary File S1.pdf]

STROBE-MR checklist

Item No.	Section	Checklist item	Page No.	Relevant text from manuscript
1	TITLE and ABSTRACT	Indicate Mendelian randomization (MR) as the study's design in the title and/or the abstract if that is a main purpose of the study	1	The title and structured abstract identify cis-QTL Mendelian randomization and regional colocalization and state the joint MR and colocalization evaluation result.
	INTRODUCTION			
2	Background	Explain the scientific background and rationale for the reported study. What is the exposure? Is a potential causal relationship between exposure and outcome plausible? Justify why MR is a helpful method to address the study question	1	The Introduction describes the randomized-trial rationale, oral exposure, candidate-gene evidence, and why MR plus colocalization is needed to constrain mechanistic inference.
3	Objectives	State specific objectives clearly, including pre-specified causal hypotheses (if any). State that MR is a method that, under specific assumptions, intends to estimate causal effects	2	The objective was to test whether any evaluated candidate gene showed both a multiplicity-controlled MR association with post-stroke mRS and regional PP.H4 ≥ 0.80.
	METHODS			
4	Study design and data sources	Present key elements of the study design early in the article. Consider including a table listing sources of data for all phases of the study. For each data source contributing to the analysis, describe the following:	2–4	The study combined summary-level cis-QTL MR, regional colocalization, external eQTL evaluation, and a predefined joint criterion for candidate-specific transcriptomic follow-up.
	a)	Setting: Describe the study design and the underlying population, if possible. Describe the setting, locations, and relevant dates, including periods of recruitment, exposure, follow-up, and data collection, when available.	2–4	Sources were GISCOME, UKB-PPP, deCODE, GTEx v10, eQTL Catalogue BLUEPRINT, and BrainSeq; all were public or controlled-access summary datasets.
	b)	Participants: Give the eligibility criteria, and the sources and methods of selection of participants. Report the sample size, and whether any power or sample size calculations were carried out prior to the main analysis	3	GISCOME included 6,165 participants overall; outcome-specific N was 6,021 for severity-adjusted ordinal mRS and mRS 0–2 versus 3–6, and 4,363 for mRS 0–1 versus 2–6. Follow-up allowed 60–190 days, with approximately 80% assessed at 3 months±2 weeks. No prospective power calculation was performed.
	c)	Describe measurement, quality control and selection of genetic variants	3	Variant quality control, allele harmonization, palindromic-variant handling, F statistics, coordinate conversion, and complete regional matching rules are reported.
	d)	For each exposure, outcome, and other relevant variables, describe methods of assessment and diagnostic criteria for diseases	3	Exposures were cis genetic proxies for candidate protein abundance or gene expression. Outcomes were within-case GISCOME functional status: severity-adjusted ordinal mRS and two binary mRS outcomes. This is not within-person recovery from baseline.
	e)	Provide details of ethics committee approval and participant informed consent, if relevant	9	Only public or controlled-access de-identified summary statistics were analysed; no participants were recruited. Ethics and consent procedures are documented by the source studies.
5	Assumptions	Explicitly state the three core IV assumptions for the main analysis (relevance, independence and exclusion restriction) as well assumptions for any additional or sensitivity analysis	3, 8	The relevance, independence, and exclusion-restriction assumptions are stated, with limitations from weak instruments, pleiotropy, index-event bias, and imperfect genetic-to-drug equivalence.

Item No.	Section	Checklist item	Page No.	Relevant text from manuscript
6	Statistical methods: main analysis	Describe statistical methods and statistics used	3–4	The manuscript defines cis instruments, Wald estimation, effect orientation, standard errors, colocalization models, priors, thresholds, and sensitivity analyses.
	a)	Describe how quantitative variables were handled in the analyses (i.e., scale, units, model)	3	GTEx NES/protein effects and GISCOME outcome alleles were harmonized; a positive MR value indicates that a genetically predicted increase corresponds to worse mRS.
	b)	Describe how genetic variants were handled in the analyses and, if applicable, how their weights were selected	3	MR used the strongest eligible cis sentinel; colocalization used all matchable variants within the standard TSS±1 Mb region rather than only the sentinel.
	c)	Describe the MR estimator (e.g. two-stage least squares, Wald ratio) and related statistics. Detail the included covariates and, in case of two-sample MR, whether the same covariate set was used for adjustment in the two samples	3	Single-variant Wald ratios and delta-method standard errors were used. Covariate adjustment and baseline-severity adjustment followed the source QTL and GISCOME models.
	d)	Explain how missing data were addressed	3–5	Complete-case, matchable summary variants were analysed without imputation; unavailable or unreliable variants were excluded according to the analysis plan.
	e)	If applicable, indicate how multiple testing was addressed	3	Eleven analysis-plan-defined gene–tissue hypotheses formed one Bonferroni family. Regional analyses used PP.H4 ≥ 0.80; prior sensitivity included $p12 = 5 \times 10^{-6}$.
7	Assessment of assumptions	Describe any methods or prior knowledge used to assess the assumptions or justify their validity	3, 7–8	Instrument F statistics, cis restriction, regional colocalization, direction checks, and an index-event-bias risk lookup were used to probe assumptions and their falsifiable limits.
8	Sensitivity analyses and additional analyses	Describe any sensitivity analyses or additional analyses performed (e.g. comparison of effect estimates from different approaches, independent replication, bias analytic techniques, validation of instruments, simulations)	3, 5	Sensitivity grids varied the region (TSS±250/500/1,000 kb), eQTL and outcome-effect priors, p12, and three outcomes. BLUEPRINT and BrainSeq provided external evaluation, and an independent script recomputed H0–H4.
9	Software and pre-registration		4	Software, inputs, versions, settings, outputs, and checks are included in reproducible scripts and the archive.
	a)	Name statistical software and package(s), including version and settings used	4	Python 3.12.13 and pysam 0.23.3 were used. Approximate-Bayes-factor colocalization was independently recomputed directly for H0–H4.
	b)	State whether the study protocol and details were pre-registered (as well as when and where)	2	The study was not prospectively registered. The prespecification status and analysis decisions are reported in Section 2.1 and the supporting information.
	RESULTS			
10	Descriptive data		4–7	Results report candidate coverage, sentinel MR, regional colocalization, the 54-tissue eGene audit, external eQTL evaluation, sensitivity analyses, and the final joint-criterion assessment.

Item No.	Section	Checklist item	Page No.	Relevant text from manuscript
	a)	Report the numbers of individuals at each stage of included studies and reasons for exclusion. Consider use of a flow diagram	4	Figure 1 reports 29 A1/A2 relations involving 19 proteins, 18 nuclear-encoded proteins assessed for QTL coverage, and 45 GTEx plus six external KMO combinations; no candidate met the joint criterion.
	b)	Report summary statistics for phenotypic exposure(s), outcome(s), and other relevant variables (e.g. means, SDs, proportions)	4–7	Tables 2–4 and Figs 2–3 report QTL/MR effects, F statistics, and PP.H4. Individual-level phenotype means and SDs are unavailable in a summary-data design.
	c)	If the data sources include meta-analyses of previous studies, provide the assessments of heterogeneity across these studies	2–3	Cross-cohort heterogeneity was inherited from GISCOME source reporting and could not be re-estimated without individual data. Outcome-specific N and the 60–190-day window are reported.
	d)	For two-sample MR:<br>i. Provide justification of the similarity of the genetic variant-exposure associations between the exposure and outcome samples<br>ii. Provide information on the number of individuals who overlap between the exposure and outcome studies	3	Exposure and outcome estimates came from separate resources. No direct overlap was known, but exact overlap counts were unavailable and small unreported overlap cannot be excluded.
11	Main results		4–7	The principal MR and colocalization results are reported with multiplicity-adjusted P values, PP.H4, directional consistency, prior sensitivity, and the joint-criterion assessment.
	a)	Report the associations between genetic variant and exposure, and between genetic variant and outcome, preferably on an interpretable scale	4–5	Tables 2–3 report gene/tissue, sentinel SNP, F statistic, MR effect, 95% CI, P value, and multiplicity decision.
	b)	Report MR estimates of the relationship between exposure and outcome, and the measures of uncertainty from the MR analysis, on an interpretable scale, such as odds ratio or relative risk per SD difference	4–5	Single-variant Wald estimates include effect size, 95% CI, and P value; positive values consistently indicate worse functional status.
	c)	If relevant, consider translating estimates of relative risk into absolute risk for a meaningful time period	Not applicable	Absolute clinical risk was not calculated because the exposures were molecular genetic proxies and the MR analyses used single-variant instruments.
	d)	Consider plots to visualize results (e.g. forest plot, scatterplot of associations between genetic variants and outcome versus between genetic variants and exposure)	2, 6–7	Fig 1 shows evidence attrition, Fig 2 the KMO regional-signal structure, and Fig 3 the PP.H4 matrix for 45 GTEx and six external combinations.
12	Assessment of assumptions		4–8	Instrument strength, regional colocalization, direction, and outcome coding were checked. All three MEGASTROKE occurrence-risk lookups were completed; HMOX1 rs2071747 was nominally associated with occurrence risk (P=0.003229), warning of index-event bias but not formally correcting it.
	a)	Report the assessment of the validity of the assumptions	4–8	Sensitivity interpretation covers instrument strength, posterior probabilities, directional tension, genetic-to-drug differences, and within-case bias.
	b)	Report any additional statistics (e.g., assessments of heterogeneity across genetic variants, such as I2, Q statistic or E-value)	4–5	F statistics and coloc H0–H4 posteriors are reported. I^2 and Cochran Q are not applicable to single-sentinel Wald estimates.

Item No.	Section	Checklist item	Page No.	Relevant text from manuscript
13	Sensitivity analyses and additional analyses		5–7	Analyses varied windows, effect priors, p12, and outcomes; used external eQTLs; independently recomputed posteriors; and completed three MEGASTROKE occurrence-risk lookups. No formal Slope-Hunter or Dudbridge correction was performed.
	a)	Report any sensitivity analyses to assess the robustness of the main results to violations of the assumptions	5	At $p12=5\times10^{-6}$, KMO whole-blood PP.H4 decreased from 0.535 to 0.366; maxima were 0.593 across GTEx and 0.347 externally. None reached 0.80. Favorable results under post hoc $p12=1\times10^{-4}$ were not used for advancement.
	b)	Report results from other sensitivity analyses or additional analyses	5, 7	BLUEPRINT monocytes and BrainSeq DLPFC provided non-GTEx evaluation; none of six combinations met PP.H4 ≥ 0.80. MEGASTROKE flagged HMOX1 occurrence risk; null KMO/OGA lookups do not prove absence of index-event bias.
	c)	Report any assessment of direction of causal relationship (e.g., bidirectional MR)	3	Reverse MR was not performed because sufficiently strong independent outcome-side instruments were unavailable; this absence is not interpreted as evidence against reverse causation.
	d)	When relevant, report and compare with estimates from non-MR analyses	8	Direct-binding and experimental pharmacology for KMO, HMOX1, and OGA are compared with the genetic and colocalization findings using separate evidence layers.
	e)	Consider additional plots to visualize results (e.g., leave-one-out analyses)	6–7	Regional posterior plots and the complete PP.H4 matrix visualize signal structure; leave-one-out analysis is not applicable to single-sentinel MR.
	DISCUSSION			
14	Key results	Summarize key results with reference to study objectives	7–8	HMOX1, OGA, and KMO lacked joint MR and regional colocalization support. Candidate-specific transcriptomic analysis was therefore not performed.
15	Limitations	Discuss limitations of the study, taking into account the validity of the IV assumptions, other sources of potential bias, and imprecision. Discuss both direction and magnitude of any potential bias and any efforts to address them	8	Limitations include a targeted rather than systematic candidate audit; unavailable formulation-matched unbound exposure; no public prospective registration; GISCOME power, timing, and ancestry constraints; within-case index-event bias; single-signal coloc; no LD-verified coloc-SuSiE; untestable single-sentinel pleiotropy; and imperfect external eQTL replication.
16	Interpretation		7–8	The Discussion relates the joint genetic-evidence result to prior pharmacology, defines the inferential scope, and identifies evidence required for future candidate-specific analyses.
	a)	Meaning: Give a cautious overall interpretation of results in the context of their limitations and in comparison with other studies	7–8	The conclusion concerns the evaluated candidate set and the available QTL and post-stroke outcome GWAS evidence.

Item No.	Section	Checklist item	Page No.	Relevant text from manuscript
	b)	Mechanism: Discuss underlying biological mechanisms that could drive a potential causal relationship between the investigated exposure and the outcome, and whether the gene-environment equivalence assumption is reasonable. Use causal language carefully, clarifying that IV estimates may provide causal effects only under certain assumptions	8	KMO biology and tension between genetic and experimental inhibition directions are discussed. Lifelong genetic perturbation is not equated with short-term multicomponent drug treatment.
	c)	Clinical relevance: Discuss whether the results have clinical or public policy relevance, and to what extent they inform effect sizes of possible interventions	1, 7–8	The randomized trial establishes formulation-level clinical relevance. The present genetic study evaluated candidate mediators and found no gene with joint MR and regional colocalization support.
17	Generalizability	Discuss the generalizability of the study results (a) to other populations, (b) across other exposure periods/timings, and (c) across other levels of exposure	8	Ancestry, timing, QTL tissue, within-case outcomes, multicomponent oral exposure, and formulation differences restrict generalizability.
	OTHER INFORMATION			
18	Funding	Describe sources of funding and the role of funders in the present study and, if applicable, sources of funding for the databases and original study or studies on which the present study is based	9	This research received no external funding. Source-study funding is reported in the original publications and database records.
19	Data and data sharing	Provide the data used to perform all analyses or report where and how the data can be accessed, and reference these sources in the article. Provide the statistical code needed to reproduce the results in the article, or report whether the code is publicly accessible and if so, where	9	The Data availability section lists public sources, derived files, checksums, and reproduction routes. The version-4 archive has reserved Zenodo DOI 10.5281/zenodo.22124922; licensed or very large original summary data are referenced and checksummed but not redistributed.
20	Conflicts of Interest	All authors should declare all potential conflicts of interest	9	The authors declare no conflicts of interest.

Manuscript: Pharmacology-Informed Human Genetic Evaluation of Panax notoginseng Saponin-Related Candidate Genes for Functional Outcome after Ischemic Stroke Using Mendelian Randomization and Colocalization

Page numbers refer to the rendered Genes submission manuscript dated 30 August 2026.

2. Skrivankova VW, Richmond RC, Woolf BAR, Davies NM, Swanson SA, VanderWeele TJ, et al. Strengthening the Reporting of Observational Studies in Epidemiology using Mendelian Randomisation (STROBE-MR): Explanation and Elaboration. BMJ. 2021;375:n2233.
