# Supplementary material for: Pharmacology-Informed Human Genetic Evaluation of Panax notoginseng Saponin-Related Candidate Genes for Functional Outcome After Ischemic Stroke Using Mendelian Randomization and Colocalization
Source: Genes (Basel). 2026 Sep 20;17(9):1155. doi: 10.3390/genes17091155 (PMC13606239; doi:10.3390/genes17091155)
Supplement: Supplementary file 1 [file genes-17-01155-s001.zip › supplementary_materials.pdf]

Supporting Information: Pharmacology-Informed Human Genetic Evaluation of *Panax notoginseng* Saponin-Related Candidate Genes for Functional Outcome After Ischemic Stroke Using Mendelian Randomization and Colocalization

Complete candidate audit, cis-QTL coverage, MR results, regional colocalization, sensitivity analyses, and reproducibility checks

Corresponding manuscript: Genes submission version, 30 August 2026

This supplement reports the complete candidate set, QTL availability, missing-data states, and all regional analyses. The candidate relations were assembled through a targeted primary-study audit. Formulation-matched unbound exposure data were unavailable for oral Xuesaitong soft capsules.

Supporting information index

Item	Content	Primary purpose
Table S1	29 A1/A2 component-protein relations	Audit pharmacology sources and exposure boundaries
Table S2	QTL coverage for 18 nuclear-encoded candidates	Document QTL coverage and analysis status
Table S3	Eleven defined eQTL sentinel analyses	Report harmonization, missingness, and multiplicity decisions
Table S4/Figure S1	Regional pQTL colocalization for HMOX1 and OGA	Complete H0–H4 and regional peaks
Table S5	KMO colocalization across 15 tissues × 3 outcomes	Complete results for 45 formal combinations
Table S6	External KMO eQTL evaluation	Six non-GTEx combinations
Table S7	KMO eGene audit in all 54 GTEx v10 tissues	Fixed tissue universe
Table S8 – S10	Sensitivity, index-event risk, and recomputation	Summarize robustness and numerical validation

All complete machine-readable results are also included in the reproducibility archive.

S1. Audit of component-protein pharmacological evidence

A1 denotes quantitative direct binding to a human protein. A2 denotes strong target-dependency or perturbation evidence when a directly comparable affinity estimate was unavailable. The 29 relations define the candidate set evaluated in this study.

Table S1. Twenty-nine A1/A2 component-protein relations

Component	Type	Target	Grade	Experiment	Direction	Potency/affinity	Exposure compatibility	Audit decision
notoR1	Parent compound	NR1I2	A2	TR-FRET competitive binding; reporter activation; human-PXR pocket mutagenesis	activate	active_concentration_range 12.5-100 uM	Possible exposure – potency incompatibility	Retain for QTL screening
notoR1	Parent compound	CKLF	A1	biolayer interferometry	bind_only	Kd 25.00 uM	Possible exposure – potency incompatibility	Retain for QTL screening
Rg1	Parent compound	CKLF	A1	MST; BLI; NanoBRET CKLF-CCR4 disruption	inhibit	Kd_MST;Kd_BLI;IC50_NanoBRET 3.42;15.52;31.64 uM	Possible exposure – potency incompatibility	Retain for QTL screening
Rb1	Parent compound	CKLF	A1	biolayer interferometry	bind_only	Kd 91.26 uM	Possible exposure – potency incompatibility	Secondary relation

Component	Type	Target	Grade	Experiment	Direction	Potency/affinity	Exposure compatibility	Audit decision
Rd	Parent compound	CKLF	A1	biolayer interferometry	bind_only	Kd 29.69 uM	Possible exposure – potency incompatibility	Retain for QTL screening
Re	Parent compound	CKLF	A1	biolayer interferometry	bind_only	Kd 66.25 uM	Possible exposure – potency incompatibility	Secondary relation
notoR1	Parent compound	MT2A	A1	surface plasmon resonance	bind_only	Kd 45.74 uM	Possible exposure – potency incompatibility	Retain for QTL screening
Rg1	Parent compound	MT2A	A1	surface plasmon resonance	bind_only	Kd 30.47 uM	Possible exposure – potency incompatibility	Retain for QTL screening
Rb1	Parent compound	MT2A	A1	surface plasmon resonance	bind_only	Kd 312.4 uM	Possible exposure – potency incompatibility	Secondary relation
Rd	Parent compound	MT2A	A1	surface plasmon resonance	bind_only	Kd 3.040 uM	Possible exposure – potency incompatibility	Retain for QTL screening

Component	Type	Target	Grade	Experiment	Direction	Potency/affinity	Exposure compatibility	Audit decision
Re	Parent compound	MT2A	A1	surface plasmon resonance	bind_only	Kd 14.32 uM	Possible exposure – potency incompatibility	Retain for QTL screening
Rg1	Parent compound	NR3C1	A2	fluorescence-polarization binding; GR reporter; GR knockdown	activate	—	Conflicting evidence and no matched PK	Retain for QTL screening
Re	Parent compound	NR3C1	A1	competitive receptor binding; antagonist and siRNA validation	activate	Kd;IC50 49.7;156.6 nM	Conflicting evidence and no matched PK	Retain for QTL screening
Rg1	Parent compound	RHOA	A1	surface plasmon resonance	bind_only	Kd 3.403 uM	Possible exposure – potency incompatibility	Retain for QTL screening
Rg1	Parent compound	CCR3	A1	surface plasmon resonance	bind_only	Kd 35.99 uM	Possible exposure – potency incompatibility	Secondary relation
Rb1	Parent compound	HMOX1	A2	SPR; MST; binding-site mutagenesis	bind_only	—	Uncertain/not calculable	Retain for QTL screening

Component	Type	Target	Grade	Experiment	Direction	Potency/affinity	Exposure compatibility	Audit decision
Rb1	Parent compound	MT-ND3	A1	SPR; complex-I bypass rescue	inhibit	Kd 0.4757 uM	Uncertain/not calculable	mechanistic only no standard cis QTL
Rb1	Parent compound	KEAP1	A2	SPR; MST; site mutagenesis	decrease_abundance	—	Uncertain/not calculable	Retain for QTL screening
Rb1	Parent compound	NCF1	A2	SPR; MST; complex-assembly assays	inhibit	—	Uncertain/not calculable	Retain for QTL screening
Rb1	Parent compound	NLRP3	A1	biolayer interferometry	inhibit	Kd 99.6 uM	Possible exposure – potency incompatibility	Secondary relation
Rd	Parent compound	NLRP3	A1	biolayer interferometry	inhibit	Kd 50.1 uM	Possible exposure – potency incompatibility	Secondary relation
Rd	Parent compound	SLC5A1	A1	SPR; CETSA	inhibit	Kd 0.68 uM	Uncertain/not calculable	Retain for QTL screening

Component	Type	Target	Grade	Experiment	Direction	Potency/affinity	Exposure compatibility	Audit decision
Rd	Parent compound	SIRT6	A2	enzyme-activity and interaction assays; liver-specific knockout	activate	—	Uncertain/not calculable	Retain for QTL screening
Rd	Parent compound	AGTR1	A2	competitive-antagonism pharmacology; receptor-pathway assays	inhibit	—	Uncertain/not calculable	Retain for QTL screening
Re	Parent compound	DNM1L	A2	target identification; site mutagenesis; genetic ablation	inhibit	—	Uncertain/not calculable	Retain for QTL screening
notoR1	Parent compound	SQSTM1	A2	microscale thermophoresis; knockout/knockdown	bind_only	—	Uncertain/not calculable	Retain for QTL screening
compound_K	Metabolite	KMO	A2	DARTS; BLI; site-directed mutagenesis	inhibit	—	Uncertain/not calculable	retain metabolite QTL screen

Component	Type	Target	Grade	Experiment	Direction	Potency/affinity	Exposure compatibility	Audit decision
compound_K	Metabolite	OGA	A1	SPR; targeted-liposome cerebral I/R validation	inhibit	Kd 3.89 uM	formulation dependent	retain metabolite QTL screen with formulation flag
20S_protopanaxadiol	Metabolite	CKM	A1	BLI; ITC; site mutagenesis	bind_only	Kd_ITC;Kd_BLI 1.9;25.3 uM	Possible exposure – potency incompatibility	retain metabolite QTL screen with exposure flag

Complete DOI, PMID, experimental system, and notes are provided in phase0_target_evidence_template.csv.

S2. QTL coverage and candidate attrition

MT-ND3 is mitochondrial encoded and was not evaluated in the standard nuclear cis-QTL framework.

Table S2. pQTL/eQTL coverage for 18 nuclear-encoded candidates

Target	Priority	cis-pQTL	GTEx v10 eQTL	Stage decision	Key limitation
NR3C1	P2	No eligible cis-pQTL	eGene in at least one defined tissue	No eligible cis-pQTL; eQTL evaluated	Not measured on either platform
SLC5A1	P1	No eligible cis-pQTL	Not detected in defined tissues	No eligible cis-pQTL; eQTL evaluated	Not measured on either platform
CKLF	P1	No eligible cis-pQTL	eGene in at least one defined tissue	No eligible cis-pQTL; eQTL evaluated	Not measured on either platform
KMO	P1	No eligible cis-pQTL	eGene in at least one defined tissue	No eligible cis-pQTL; eQTL evaluated	Not measured on either platform
NR1I2	P2	No eligible cis-pQTL	eGene in at least one defined tissue	No eligible cis-pQTL; eQTL evaluated	Not measured on either platform
MT2A	P2	No eligible cis-pQTL	eGene in at least one defined tissue	No eligible cis-pQTL; eQTL evaluated	Not measured on either platform
HMOX1	P2	UKB/deCODE	eGene in at least one defined tissue	Included in pQTL analysis	Exposure – potency relation remains uncertain

Target	Priority	cis-pQTL	GTEx v10 eQTL	Stage decision	Key limitation
KEAP1	P2	No eligible cis-pQTL	eGene in at least one defined tissue	No eligible cis-pQTL; eQTL evaluated	Measured by deCODE but no cis-pQTL
NCF1	P2	deCODE	eGene in at least one defined tissue	Included in pQTL analysis	Single platform; pseudogene/alignment complexity
SIRT6	P2	No eligible cis-pQTL	eGene in at least one defined tissue	No eligible cis-pQTL; eQTL evaluated	Not measured on either platform
AGTR1	P2	No eligible cis-pQTL	eGene in at least one defined tissue	No eligible cis-pQTL; eQTL evaluated	Not measured on either platform
DNM1L	P2	No eligible cis-pQTL	eGene in at least one defined tissue	No eligible cis-pQTL; eQTL evaluated	Not measured on either platform
SQSTM1	P2	No eligible cis-pQTL	eGene in at least one defined tissue	No eligible cis-pQTL; eQTL evaluated	Measured by deCODE but no cis-pQTL
RHOA	P3	No eligible cis-pQTL	eGene in at least one defined tissue	No eligible cis-pQTL; eQTL evaluated	Measured by deCODE but no cis-pQTL
NLRP3	P3	No eligible cis-pQTL	eGene in at least one defined tissue	No eligible cis-pQTL; eQTL evaluated	Not measured on either platform

Target	Priority	cis-pQTL	GTEx v10 eQTL	Stage decision	Key limitation
CCR3	P3	No eligible cis-pQTL	eGene in at least one defined tissue	No eligible cis-pQTL; eQTL evaluated	Not measured on either platform
OGA	P3	UKB	Not detected in defined tissues	Included in pQTL analysis	Single platform; dependent on targeted-liposome formulation
CKM	P3	No eligible cis-pQTL	eGene in at least one defined tissue	No eligible cis-pQTL; eQTL evaluated	deCODE assay excluded; not measured in UKB

S3. CKLF/KMO/MT2A eQTL sentinel analyses

Table S3. Eleven analysis-plan-defined gene–tissue hypotheses

Gene	Tissue	SNP	F	GISCOME	MR β	SE	P	Adjusted P	Decision
CKLF	Brain caudate	rs12599230	16.41	Missing	—	—	—	—	Not estimable
KMO	Whole blood	rs3819976	36.93	Harmonized	-0.906	0.314	0.0039	0.043	Selected for regional colocalization
KMO	Brain caudate	rs73139034	17.87	Missing	—	—	—	—	Not estimable
KMO	Brain cortex	rs6679186	26.31	Harmonized	-0.667	0.240	0.0054	0.059	Not selected for colocalization
KMO	Brain frontal cortex BA9	rs6681649	28.80	Harmonized	-0.552	0.204	0.0067	0.074	Not selected for colocalization
KMO	Brain nucleus accumbens	rs72766100	18.54	Harmonized	-0.157	0.127	0.2149	1.000	Not selected for colocalization
KMO	Coronary artery	rs4659998	20.53	Missing	—	—	—	—	Not estimable
MT2A	Whole blood	rs1580833	42.84	Harmonized	-0.464	0.253	0.0671	0.738	Not selected for colocalization
MT2A	Brain caudate	rs79836464	17.33	Missing	—	—	—	—	Not estimable

Gene	Tissue	SNP	F	GISCOME	MR β	SE	P	Adjusted P	Decision
MT2A	Aorta	rs150270963	16.89	Harmonized	0.588	0.373	0.1150	1.000	Not selected for colocalization
MT2A	Tibial artery	rs72791653	16.19	Harmonized	0.051	0.403	0.8996	1.000	Not selected for colocalization

MR effects were oriented so that a positive value indicated worse mRS. Adjusted P values use Bonferroni correction across 11 hypotheses.

S4. Regional pQTL colocalization for HMOX1 and OGA

Table S4. Primary regional posterior probabilities for HMOX1 and OGA

Gene	nSNP	PP.H1	PP.H2	PP.H3	PP.H4	H4/(H3+H4)	pQTL lead	Outcome lead	Criterion met
HMOX1	7144	0.631	0.000	0.336	0.033	0.090	rs2071747	rs132703	No
OGA	4107	0.789	0.000	0.183	0.027	0.130	rs3781299	rs149647905	No

Primary priors were $p1 = p2 = 1 \times 10^{-4}$ and $p12 = 1 \times 10^{-5}$. Sensitivity analysis at $p12 = 5 \times 10^{-6}$ yielded PP.H4 = 0.021 for HMOX1 and 0.019 for OGA; neither result met the PP.H4 ≥ 0.80 criterion.

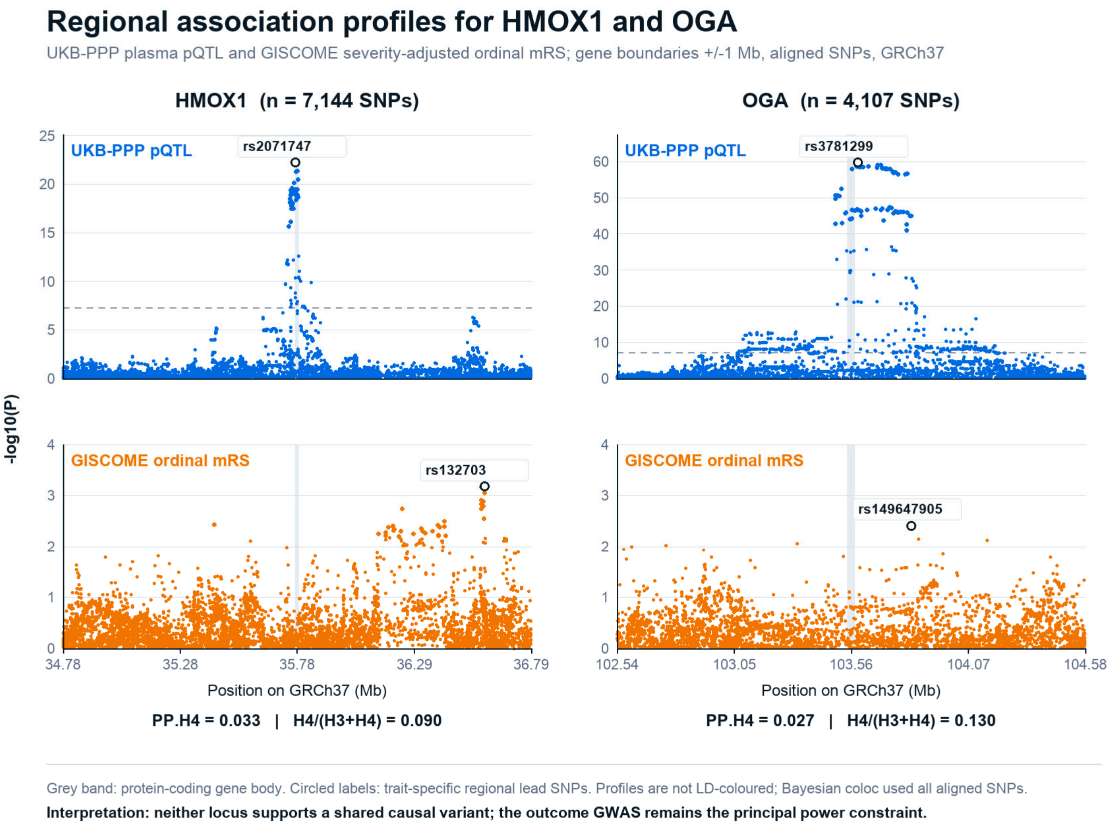

Figure S1. Regional association profiles for HMOX1 and OGA pQTLs and GISCOME severity-adjusted ordinal mRS. Neither pQTL peak shared the regional outcome signal with high PP.H4.

S5. Forty-five formal KMO colocalization combinations in the standard cis window

The fixed KMO window was TSS±1 Mb (GRCh38 chr1:240,532,134–242,532,134). The table reports all 15 GTEx v10 eGene tissues and three GISCOME outcomes; tissues were not selected by PP.H4 ranking.

Table S5. KMO regional colocalization across 15 tissues and three outcomes

Tissue	Outcome	nSNP	PP.H1	PP.H3	PP.H4	H4/(H3+H4)	eQTL lead	Outcome lead
Coronary artery	ordinal	6867	0.448	0.263	0.019	0.068	1:240848034:T:C	rs66497254
Coronary artery	mRS 0–2	6859	0.458	0.252	0.020	0.072	1:240848034:T:C	rs61825624
Coronary artery	mRS 0–1	7661	0.447	0.266	0.024	0.083	1:240848034:T:C	rs78533357
Brain caudate	ordinal	6867	0.243	0.143	0.023	0.141	rs12034492	rs66497254
Brain caudate	mRS 0–2	6859	0.249	0.137	0.023	0.143	rs12034492	rs61825624
Brain caudate	mRS 0–1	7661	0.259	0.154	0.036	0.191	rs12034492	rs78533357
Brain cortex	ordinal	6867	0.281	0.165	0.460	0.736	rs6679186	rs66497254
Brain cortex	mRS 0–2	6859	0.433	0.239	0.188	0.441	rs6679186	rs61825624
Brain cortex	mRS 0–1	7661	0.416	0.247	0.201	0.449	rs6679186	rs78533357
Brain frontal cortex BA9	ordinal	6867	0.288	0.169	0.479	0.740	rs6681649	rs66497254
Brain frontal cortex BA9	mRS 0–2	6859	0.478	0.263	0.155	0.371	rs6681649	rs61825624

Tissue	Outcome	nSNP	PP.H1	PP.H3	PP.H4	H4/(H3+H4)	eQTL lead	Outcome lead
Brain frontal cortex BA9	mRS 0–1	7661	0.432	0.257	0.215	0.456	rs6681649	rs78533357
Brain nucleus accumbens	ordinal	6867	0.300	0.176	0.031	0.148	rs72766100	rs66497254
Brain nucleus accumbens	mRS 0–2	6859	0.308	0.169	0.028	0.143	rs72766100	rs61825624
Brain nucleus accumbens	mRS 0–1	7656	0.313	0.186	0.023	0.108	rs72766100	rs78533357
EBV-transformed lymphocytes	ordinal	6867	0.297	0.174	0.528	0.752	rs3819976	rs66497254
EBV-transformed lymphocytes	mRS 0–2	6859	0.535	0.295	0.169	0.365	rs3819976	rs61825624
EBV-transformed lymphocytes	mRS 0–1	7661	0.473	0.281	0.245	0.466	rs3819976	rs78533357
Sigmoid colon	ordinal	6867	0.224	0.132	0.021	0.136	rs116429002	rs66497254
Sigmoid colon	mRS 0–2	6859	0.229	0.126	0.022	0.148	rs116429002	rs61825624
Sigmoid colon	mRS 0–1	7661	0.242	0.144	0.020	0.120	rs142075872	rs78533357
Transverse colon	ordinal	6867	0.235	0.138	0.026	0.159	rs12041617	rs66497254

Tissue	Outcome	nSNP	PP.H1	PP.H3	PP.H4	H4/(H3+H4)	eQTL lead	Outcome lead
Transverse colon	mRS 0–2	6859	0.241	0.133	0.019	0.127	rs12041617	rs61825624
Transverse colon	mRS 0–1	7661	0.255	0.152	0.020	0.118	rs12041617	rs78533357
Esophagus mucosa	ordinal	6867	0.238	0.140	0.021	0.133	rs72768077	rs66497254
Esophagus mucosa	mRS 0–2	6859	0.244	0.134	0.018	0.120	rs72768077	rs61825624
Esophagus mucosa	mRS 0–1	7661	0.253	0.150	0.019	0.114	rs72768077	rs78533357
Heart atrial appendage	ordinal	6867	0.268	0.157	0.021	0.118	rs112835210	rs66497254
Heart atrial appendage	mRS 0–2	6859	0.274	0.151	0.019	0.113	rs112835210	rs61825624
Heart atrial appendage	mRS 0–1	7661	0.284	0.169	0.019	0.102	rs78545553	rs78533357
Tibial nerve	ordinal	6867	0.612	0.359	0.029	0.075	rs12083705	rs66497254
Tibial nerve	mRS 0–2	6859	0.584	0.322	0.094	0.226	rs12083705	rs61825624
Tibial nerve	mRS 0–1	7661	0.609	0.362	0.029	0.075	rs12083705	rs78533357
Spleen	ordinal	6867	0.294	0.173	0.531	0.755	rs3819976	rs66497254
Spleen	mRS 0–2	6859	0.532	0.293	0.170	0.368	rs3819976	rs61825624

Tissue	Outcome	nSNP	PP.H1	PP.H3	PP.H4	H4/(H3+ H4)	eQTL lead	Outcome lead
Spleen	mRS 0–1	7661	0.484	0.288	0.225	0.439	rs75928083	rs78533357
Testis	ordinal	6867	0.591	0.347	0.054	0.134	rs4660103	rs66497254
Testis	mRS 0–2	6859	0.608	0.335	0.049	0.128	rs4660103	rs61825624
Testis	mRS 0–1	7661	0.607	0.361	0.024	0.062	rs4660103	rs78533357
Thyroid	ordinal	6867	0.194	0.114	0.096	0.458	rs10926505	rs66497254
Thyroid	mRS 0–2	6859	0.212	0.117	0.037	0.240	rs10926505	rs61825624
Thyroid	mRS 0–1	7661	0.228	0.136	0.040	0.229	rs77045116	rs78533357
Whole blood	ordinal	6867	0.293	0.171	0.535	0.757	rs3819976	rs66497254
Whole blood	mRS 0–2	6859	0.533	0.294	0.173	0.370	rs3819976	rs61825624
Whole blood	mRS 0–1	7661	0.469	0.279	0.251	0.474	rs3819976	rs78533357

All combinations had PP.H4<0.80. Complete H0–H4 posterior probabilities, priors, and shared-variant probabilities are provided in kmo_standard_cis_all_outcomes_coloc_summary.csv.

S6. External KMO eQTL evaluation

Table S6. Six BLUEPRINT and BrainSeq colocalization combinations

Dataset	n	Outcome	nSNP	PP.H1	PP.H3	PP.H4	eQTL lead	Outcome lead	Criterion met
BLUEPRINT monocyte	191	ordinal	6871	0.246	0.145	0.045	rs61737760	rs66497254	No
BLUEPRINT monocyte	191	mRS 0–2	6863	0.253	0.138	0.044	rs61737760	rs61825624	No
BLUEPRINT monocyte	191	mRS 0–1	7004	0.265	0.138	0.026	rs61737760	rs78533357	No
BrainSeq DLPFC	479	ordinal	6373	0.250	0.134	0.247	rs3819976	rs66497254	No
BrainSeq DLPFC	479	mRS 0–2	6374	0.317	0.158	0.066	rs3819976	rs61825624	No
BrainSeq DLPFC	479	mRS 0–1	7126	0.317	0.173	0.092	rs3819976	rs74151809	No

The strongest eQTLs fell just short of the regional Bonferroni threshold; concordant direction is therefore described only as limited directional consistency.

S7. Complete GTEx v10 eGene-tissue audit

Table S7. KMO eGene status in all 54 GTEx v10 tissues

Tissue	n	eGene	Significant pairs	lead SNP	lead P	lead NES
Adipose Subcutaneous	711	No	0	—	—	—
Adipose Visceral Omentum	584	No	0	—	—	—
Adrenal Gland	295	No	0	—	—	—
Artery Aorta	472	No	0	—	—	—
Artery Coronary	268	Yes	4	rs4659998	5.88e-06	0.199
Artery Tibial	689	No	0	—	—	—
Bladder	77	No	0	—	—	—
Brain Amygdala	180	No	0	—	—	—
Brain Anterior cingulate cortex BA24	233	No	0	—	—	—
Brain Caudate basal ganglia	298	Yes	2	rs73139034	2.37e-05	-1.079
Brain Cerebellar Hemisphere	276	No	0	—	—	—
Brain Cerebellum	264	No	0	—	—	—

Tissue	n	eGene	Significant pairs	lead SNP	lead P	lead NES
Brain Cortex	268	Yes	51	rs6679186	2.91e-07	-0.581
Brain Frontal Cortex BA9	268	Yes	46	rs6681649	8.03e-08	-0.629
Brain Hippocampus	254	No	0	—	—	—
Brain Hypothalamus	256	No	0	—	—	—
Brain Nucleus accumbens basal ganglia	284	Yes	3	rs72766100	1.66e-05	0.352
Brain Putamen basal ganglia	253	No	0	—	—	—
Brain Spinal cord cervical c-1	203	No	0	—	—	—
Brain Substantia nigra	183	No	0	—	—	—
Breast Mammary Tissue	511	No	0	—	—	—
Cells Cultured fibroblasts	649	No	0	—	—	—
Cells EBV-transformed lymphocytes	326	Yes	53	rs3819976	1.86e-10	-0.684
Cervix Ectocervix		No	0	—	—	—

Tissue	n	eGene	Significant pairs	lead SNP	lead P	lead NES
Cervix Endocervix		No	0	—	—	—
Colon Sigmoid	418	Yes	1	rs116712055	1.21e-06	-1.178
Colon Transverse	478	Yes	2	rs68166816	1.24e-05	-0.221
Esophagus Gastroesophageal Junction	403	No	0	—	—	—
Esophagus Mucosa	614	Yes	1	rs72768077	3.94e-05	-0.236
Esophagus Muscularis	561	No	0	—	—	—
Fallopian Tube		No	0	—	—	—
Heart Atrial Appendage	460	Yes	5	rs78545553	5.55e-06	-0.765
Heart Left Ventricle	450	No	0	—	—	—
Kidney Cortex	103	No	0	—	—	—
Kidney Medulla		No	0	—	—	—
Liver	261	No	0	—	—	—
Lung	601	No	0	—	—	—
Minor Salivary Gland	180	No	0	—	—	—

Tissue	n	eGene	Significant pairs	lead SNP	lead P	lead NES
Muscle Skeletal	816	No	0	—	—	—
Nerve Tibial	667	Yes	48	rs12083705	1.39e-56	1.256
Ovary	192	No	0	—	—	—
Pancreas	362	No	0	—	—	—
Pituitary	311	No	0	—	—	—
Prostate	281	No	0	—	—	—
Skin Not Sun Exposed Suprapubic	649	No	0	—	—	—
Skin Sun Exposed Lower leg	751	No	0	—	—	—
Small Intestine Terminal Ileum	206	No	0	—	—	—
Spleen	277	Yes	62	rs75928083	9.05e-11	-0.955
Stomach	407	No	0	—	—	—
Testis	413	Yes	25	rs34909646	5.85e-16	-0.388
Thyroid	681	Yes	5	rs77045116	4.17e-05	-0.663
Uterus	152	No	0	—	—	—
Vagina	170	No	0	—	—	—

Tissue	n	eGene	Significant pairs	lead SNP	lead P	lead NES
Whole Blood	800	Yes	29	rs3819976	1.22e-09	-0.406

Only the 15 tissues meeting the eGene FDR criterion entered formal regional analysis; tissues were not selected retrospectively using GISCOME results.

S8. Sensitivity to priors, windows, and external datasets

Table S8. Summary of colocalization sensitivity analyses

Analysis layer	Combinations/grid size	Setting with maximum	Maximum PP.H4	Criterion assessment
Formal GTEx primary setting	45	TSS±1 Mb；p12=1e-5	0.535	Not met
Conventional GTEx sensitivity	4860	Brain_Cortex / ordinal_adjusted / tss_plus_minus_250kb	0.744	Not met
Complete GTEx prior grid	8100	p12=0.0001 (equal to p1/p2)	0.967	PP.H4 ≥ 0.80 only at p12 = 1×10^{-4}
Formal external combinations	6	Same TSS±1 Mb window and primary priors	0.247	Not met
Conventional external sensitivity	216	BrainSeq_DLPFC / ordinal_adjusted	0.515	Not met
Complete external prior grid	360	p12=0.0001 (equal to p1/p2)	0.914	PP.H4 ≥ 0.80 only at p12 = 1×10^{-4}
Robust GTEx shared-variant prior	1,620	p12=5×10^{-6}；45 tissue-outcome combinations × 3 windows × 3 eQTL-effect SDs × 4 outcome-effect SDs	0.593	Not met

Analysis layer	Combinations/grid size	Setting with maximum	Maximum PP.H4	Criterion assessment
Robust re-evaluation of the primary KMO analysis-plan combination	1	Whole blood × ordinal；TSS±1 Mb；$p12=5\times10^{-6}$	0.366	Not met
Robust external-eQTL shared-variant prior	Combination grid	BrainSeq DLPFC × ordinal ；$p12=5\times10^{-6}$	0.347	Not met
Robust HMOX1/OGA shared-variant prior	2 genes	$p12=5\times10^{-6}$	0.021/0.019	Neither met the criterion

At $p12 = 5 \times 10^{-6}$, PP.H4 was 0.366 for the primary KMO whole-blood comparison, with maxima of 0.593 across the GTEx grid and 0.347 across the external eQTL grid. Values at $p12 = 1 \times 10^{-4}$ are reported as prior-sensitivity results.

S9. Index-event-bias risk lookup

Table S9. Candidate instruments and ischemic-stroke occurrence risk in European ancestry

Target	Instrument SNP	Risk GWAS	EA	OA	β	SE	P	Status
OGA	rs3781299	MEGASTROKE European AIS (GCST006908)	A	C	0.0121	0.0109	0.2667	Completed
HMOX1	rs2071747	MEGASTROKE European AIS (GCST006908)	C	G	0.0644	0.0219	0.003229	Completed
KMO	rs3819976	MEGASTROKE European AIS (GCST006908)	C	T	-0.0128	0.0337	0.7046	Completed

OGA and KMO instruments were not associated with ischemic-stroke occurrence. HMOX1 rs2071747 was associated with occurrence risk (P = 0.00323), identifying a potential source of index-event bias in the within-case outcome analysis.

S10. Independent recomputation and input quality control

Table S10. Numerical validation and data integrity

Check	Scope	Result	Conclusion
Direct posterior recomputation for formal GTEx combinations	45/45	maximum absolute difference 6.94e-15	Passed
Direct posterior recomputation for external-data combinations	6/6	maximum absolute difference 6.66e-16	Passed
KMO standard-window position and variant uniqueness	45/45	All variants were unique and within the defined window	Passed
Cross-outcome tissue-variant eQTL consistency	102,510 pairs	0 numerical discrepancies	Passed
GTEx dynamic-interface completeness	15Tissue	Brain nucleus accumbens5 API records missing; zero elsewhere; total API errors 5	Complete-case handling

Validation files in the archive document outcome-specific sample sizes, allele orientation, variant matching, posterior recomputation, prior sensitivity, and MEGASTROKE occurrence-risk lookups.

S11. Data and code archive

The archive does not redistribute licensed or very large original summary statistics. It provides source manifests, analysis code, parameters, checksums, and derived result tables. The Zenodo draft has reserved DOI https://doi.org/10.5281/zenodo.22124922.

Table S11. Key machine-readable files

File	Purpose
phase0_target_evidence_template.csv	Master table of 29 pharmacological relations
phase1_qtl_coverage_manifest.csv	QTL coverage audit for 18 candidates
prespecified_11_eqtl_hypotheses_status.csv	Status of 11 defined analyses
hmox1_oga_coloc_abf_summary.csv	HMOX1/OGA regional posterior probabilities
kmo_standard_cis_all_outcomes_coloc_summary.csv	45 formal KMO colocalization combinations
kmo_standard_cis_all_outcomes_coloc_sensitivity.csv	8,100-row KMO sensitivity grid
kmo_independent_eqtl_replication_coloc_summary.csv	6 external eQTL combinations
index_event_bias_risk_lookup.csv	Completeness of ischemic-stroke occurrence-risk lookup
kmo_standard_cis_analysis_validation.json	Independent posterior recomputation
MEGASTROKE_index_event_three_variants.csv	Redistributable derived rows for occurrence-risk lookups of three instruments

The archive contains the analysis code, derived outputs, validation files, and generation scripts.

Core analysis commands

```
python code/eqtl/run_eqtl_sentinel_mr.py
python code/eqtl/run_kmo_standard_cis_all_outcomes_coloc.py
```

```
python code/eqtl/run_kmo_independent_eqtl_replication.py
```

Input sources, execution order, and checksums are described in README_REPRODUCIBILITY.md and MANIFEST.tsv.
